# Free Flap Fat Volume is Not Associated With Recurrence or Wound Complications in Oral Cancer

**DOI:** 10.1002/oto2.46

**Published:** 2023-03-28

**Authors:** Andre J. Burnham, Jaime Wicks, Kristen L. Baugnon, Mark W. El‐Deiry, Nicole C. Schmitt

**Affiliations:** ^1^ Department of Otolaryngology–Head and Neck Surgery Emory University School of Medicine Atlanta Georgia USA; ^2^ Department of Radiology Emory University School of Medicine Atlanta Georgia USA; ^3^ Head and Neck Program Winship Cancer Institute at Emory University Georgia Atlanta USA

**Keywords:** adipose stem cells, adipose tissue, free flap, head and neck cancer, recurrence

## Abstract

**Objective:**

Adipose stem cells (ASCs) have been shown in many preclinical studies to be potent suppressors of the immune system. Prior studies suggest that ASCs may promote cancer progression and wound healing. However, clinical studies investigating the effects of native, or fat‐grafted adipose tissue on cancer recurrence have generated mixed results. We investigated whether adipose content in reconstructive free flaps for oral squamous cell carcinoma (OSCC) is associated with disease recurrence and/or reduction in wound complications.

**Study Design:**

Retrospective chart review.

**Setting:**

Academic medical center.

**Methods:**

We performed a review of 55 patients undergoing free flap reconstruction for OSCC over a 14‐month period. Using texture analysis software, we measured the relative free flap fat volume (FFFV) in postoperative computed tomography scans and compared fat volume with patient survival, recurrence, and wound healing complications.

**Results:**

We report no difference in mean FFFV between patients with or without recurrence: 13.47 cm^3^ in cancer‐free survivors and 17.99 cm^3^ in cases that recurred (*p* = .56). Two‐year recurrence‐free survival in patients with high and low FFFV was 61.0% and 59.1%, respectively (*p* = .917). Although only 9 patients had wound healing complications, we found no trend in the incidence of wound healing complications between patients with high versus low FFFV.

**Conclusion:**

FFFV is not associated with recurrence or wound healing in patients undergoing free flap reconstruction for OSCC, suggesting adipose content should not be of concern to the reconstructive surgeon.

Head and neck squamous cell carcinoma (HNSCC) is associated with significant morbidity and mortality, with 5‐year survival estimated at approximately 55% to 66%.^1^ The standard of care for HNSCC includes surgical resection, radiation therapy, and/or chemotherapy. Depending on the tumor location and extent, microvascular free flap reconstruction is often necessary in order to preserve anatomic functionality and cosmetic appearance. Free flaps may be harvested from a variety of sites, including the radial forearm, latissimus dorsi, fibula, thigh, and scapula. Free flaps usually contain a significant amount of adipose tissue, which is inherently rich in adipose mesenchymal stromal cells, also known as adipose stem cells (ASCs).

ASCs are multipotent progenitor cells ubiquitously present in adipose tissue.^2^ ASCs are widely recognized to possess potent immunomodulatory activity and may contribute to wound healing and repair of inflamed and damaged tissues.^3^, ^4^, ^5^ Interestingly, preclinical studies have suggested that adipose tissue and ASCs native to adipose tissue may play a role in tumor progression in vitro and in vivo by suppressing antitumor T cell cytotoxicity, promoting angiogenesis, and other paracrine interactions.^6^, ^7^, ^8^, ^9^ Moreover, recent laboratory studies have suggested that ASCs may increase angioinvasion, migration, metastasis, and tumor cell proliferation in head and neck cancer.^10^, ^11^

Clinical studies have also suggested that adipose tissue, and presumably ASCs, may promote tumor recurrence following ablative and reconstructive surgery. Several studies have reported no significant difference in tumor recurrence in patients that underwent fat grafting following tumor resection (notably in breast cancer),^12^, ^13^, ^14^ while other studies have shown an increased risk of breast cancer recurrence following autologous fat grafting.^15^, ^16^ Given that clinical studies have generated mixed results in breast cancer, and basic science research continues to investigate the role ASCs in cancer progression, there is a need for additional clinical research studying the risk of adipose‐rich free flaps and fat grafting in various malignancies, including head and neck cancer. The existing literature raises concern that perhaps the head and neck reconstructive surgeon may need to consider adipose content when choosing and designing a free flap for oral cancer reconstruction.

Here, we retrospectively evaluated oral squamous cell carcinoma (OSCC) patients that underwent free flap reconstruction immediately following ablative surgery to test the hypothesis that adipose content of free flap tissue is associated with disease recurrence. Specifically, we measured the relative fat content in the, respectively, computed tomography (CT) scans and compared flap fat content with patient survival and recurrence outcomes. We also recorded wound healing complications to determine whether the fat content of free flaps is associated with improved wound healing.

## Materials and Methods

### Study Population

This study was approved by the Emory University Institutional Review Board (STUDY00001945). A waiver of informed consent was granted for this retrospective chart review. The electronic database maintained by the Emory University School of Medicine Department of Otolaryngology was queried for patients with a diagnosis of OSCC undergoing surgical ablation and free flap reconstruction between January 2017 and February 2018, with postoperative CT scans obtained between 6‐ and 12‐months following surgery. Medical charts, including operative notes, pathology reports, and radiographic imaging, were reviewed and data were extracted.

### Patient Data Collection

Patient demographic data collected included age, sex, and race. The following disease characteristics were included to account for confounders: tumor site, tumor, node, and metastasis stage, grade, surgical resection margins, perineural invasion, lymphovascular invasion, and extracapsular extension. Other comorbid factors at the time of surgery were quantified using the Charlson comorbidity index, and smoking status. Details on adjuvant therapy and flap type were also recorded. Outcomes measured included 2‐year recurrence‐free survival (RFS), overall survival (OS), and wound healing complications including fistula, abscess, and dehiscence.

### Image Analysis

Free flap fat volume (FFFV) was measured using the radiographic texture analysis software LIFEx (version 7.1.0). Neck CT DICOMS were loaded into LIFEx and using default program parameters, regions of interest were manually drawn in coronal, axial, and sagittal planes around free flap subcutaneous fat. Using LIFEx texture analysis, 3‐dimensional volumes of interest (VOI) were generated to include flap adipose tissue and exclude tissue outside adipose margins. Scans for which adipose tissue could not be identified (eg, flaps composed of muscle) were assigned VOIs of 0 cm^3^. The median FFFV in the patient population was 4.97 cm^3^, and for the purposes of data analysis, patients with FFFV less than 5 cm^3^ were assigned to the low FFFV group, while those greater than 5 cm^3^ were assigned to the high FFFV group.

### Statistical Analyses

Figures were generated in GraphPad/Prism (version 8.2). Unpaired 2‐tailed student *t* tests were used to determine the statistical significance of patient FFFV between recurrence and nonrecurrence groups and between patients with and without wound healing complications. To compare the incidence of wound healing complications at FFFV above and below the median (5 cm^3^), Fisher's exact test was used. Survival data were graphed using Kaplan‐Meier methods and analyzed by the log‐rank (Mantel‐Cox) test.

## Results

### Patient Characteristics

Prior to extracting data from the electronic medical record, we ran a sample size estimate. Recurrence occurs within 2 years in approximately 40% of patients with advanced (T3/T4) oral cancer.^17^ We determined that a difference in recurrence of 25% would be sufficiently meaningful to consider reevaluating our clinical practices and free flap selection. Assuming 30% recurrence in patients with FFFV below the median and 55% recurrence in patients with FFFV above the median, with 80% power and *α* of .05, we determined that 120 subjects would be needed to detect a statistically significant difference of 25%. However, given the labor‐intensive process of measuring fat content on CT scans, we performed an interim futility analysis after the first 55 patients. When we did not observe any associations during the interim analyses, we elected not to review additional charts.

Patient demographic data, disease characteristics, free flap type, and comorbidities are summarized in Table 1. As might be expected, most patients had advanced (T3/T4) tumors at the time of surgery. Radial forearm and fibula flaps were used most commonly.

**Table 1:** Demographics and Disease Characteristics of Patients With OSCC Undergoing Free Flap Reconstruction

Characteristic	n = 55
Patient demographics	
Age, mean (SD)	61.4 (11.3)
Male, n (%)	39 (70.9)
Female, n (%)	16 (29.1)
White, n (%)	38 (69.1)
Black, n (%)	11 (20.0)
Asian, n (%)	5 (9.1)
Charlson comorbidity index, n (%)	
2	43 (78.2)
3	7 (12.7)
4	3 (5.5)
5	2 (3.6)
Smoking status, n (%)	
Never smoker	27 (49.1)
Current smoker	13 (23.6)
Reformed smoker >15 y	2 (3.6)
Reformed smoker ≤15 y	13 (23.6)
Sites involved by tumor, n (%)	
Tongue	14 (25.5)
RMT	13 (23.6)
FOM	16 (29.1)
Buccal	8 (14.5)
Lower gingiva/lip	3 (5.5)
Tumor grade, n (%)	
G0	1 (1.8)
G1	5 (9.1)
G2	46 (83.6)
G3	3 (5.5)
T stage, n (%)	
1	6 (10.9)
2	12 (21.8)
3	4 (7.3)
4	2 (3.6)
4a	25 (45.5)
4b	6 (10.9)
N stage, n (%)	
0	25 (45.5)
1	6 (10.9)
2a	1 (1.8)
2b	12 (21.8)
2c	6 (10.9)
3b	4 (7.3)
NA	1 (1.8)
Tissue invasion (other), n (%)	
PNI	28 (50.9)
LVI	20 (36.4)
ECE	21 (38.2)
Positive margins	7 (12.7)
Type of flap, n (%)	
Radial	20 (36.4)
Fibula	21 (38.2)
Scapula	11 (20.0)
Other	3 (5.5)

Abbreviations: ECE, extracapsular extension; FOM, floor of mouth; LVI, lymphovascular invasion; NA, not applicable; OSCC, oral squamous cell carcinoma; PNI, perineural invasion; RMT, retromolar trigone; SD, standard deviation.

### Recurrence and Survival

**Figure 1:**
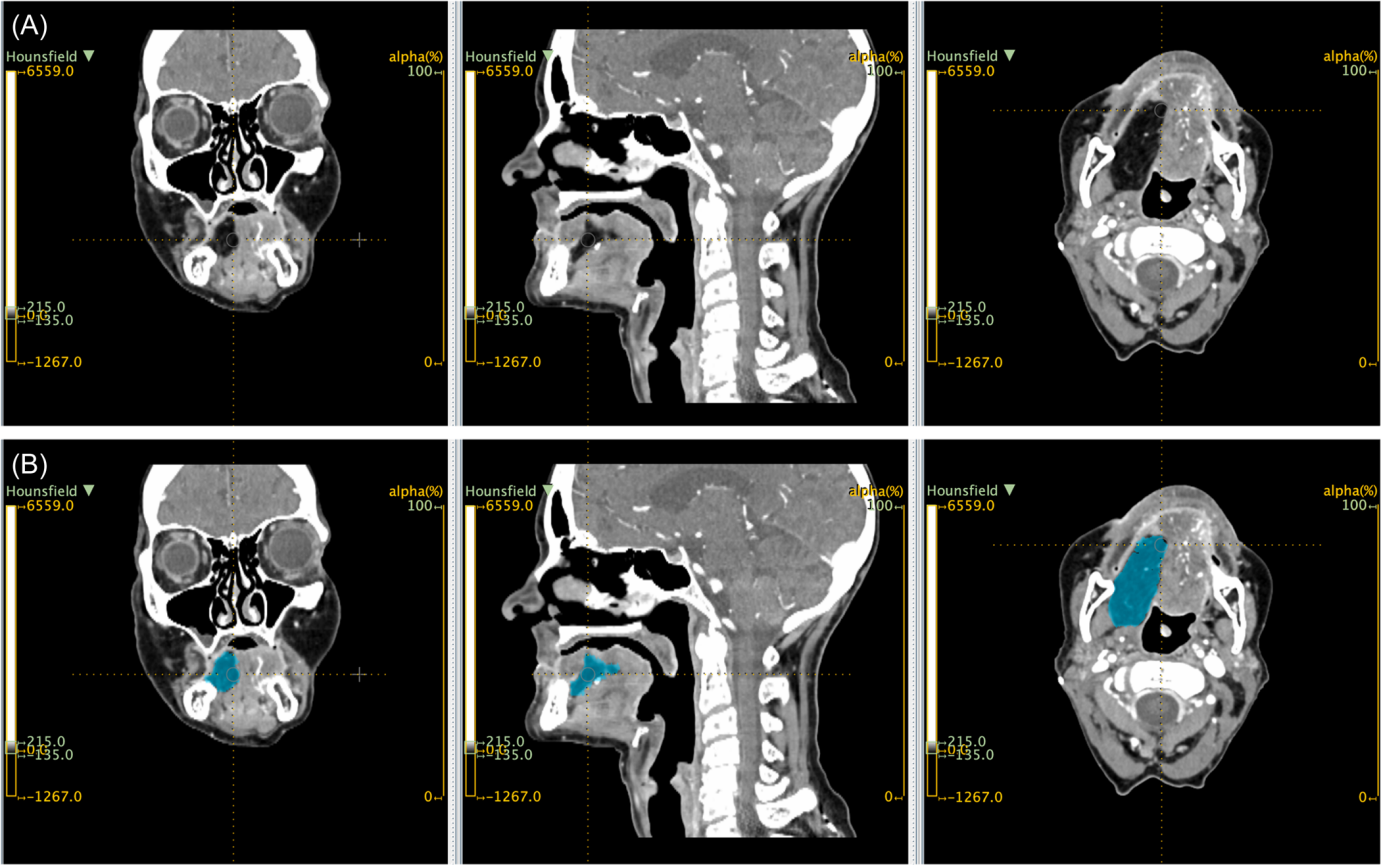
Representative CT imaging imported into LIFEx showing planes of coronal, sagittal and axial views (A) before texture analysis, and (B) after 3D ROI generation of free flap fat volume. 3D, 3‐dimensional; CT, computed tomography; ROI, regions of interest.

**Figure 2:**
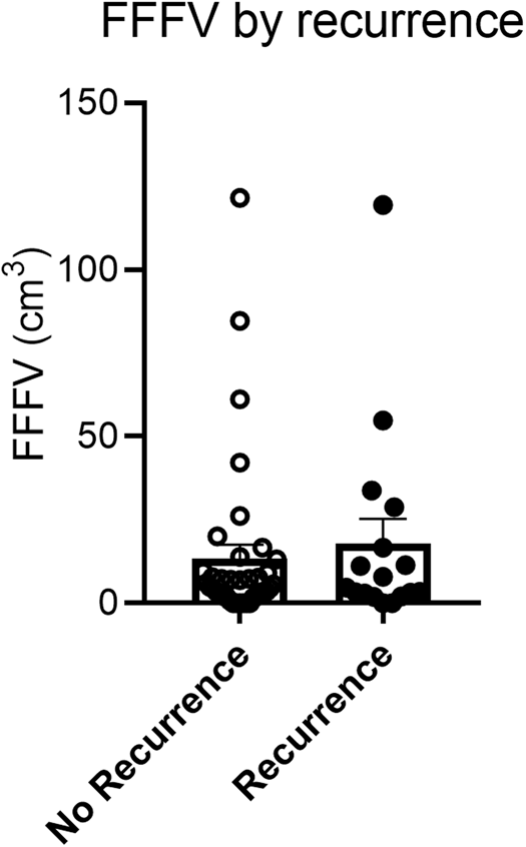
Free flap fat volume (FFFV) did not differ between cases with versus without recurrence. Data are mean + standard error of mean.

**Figure 3:**
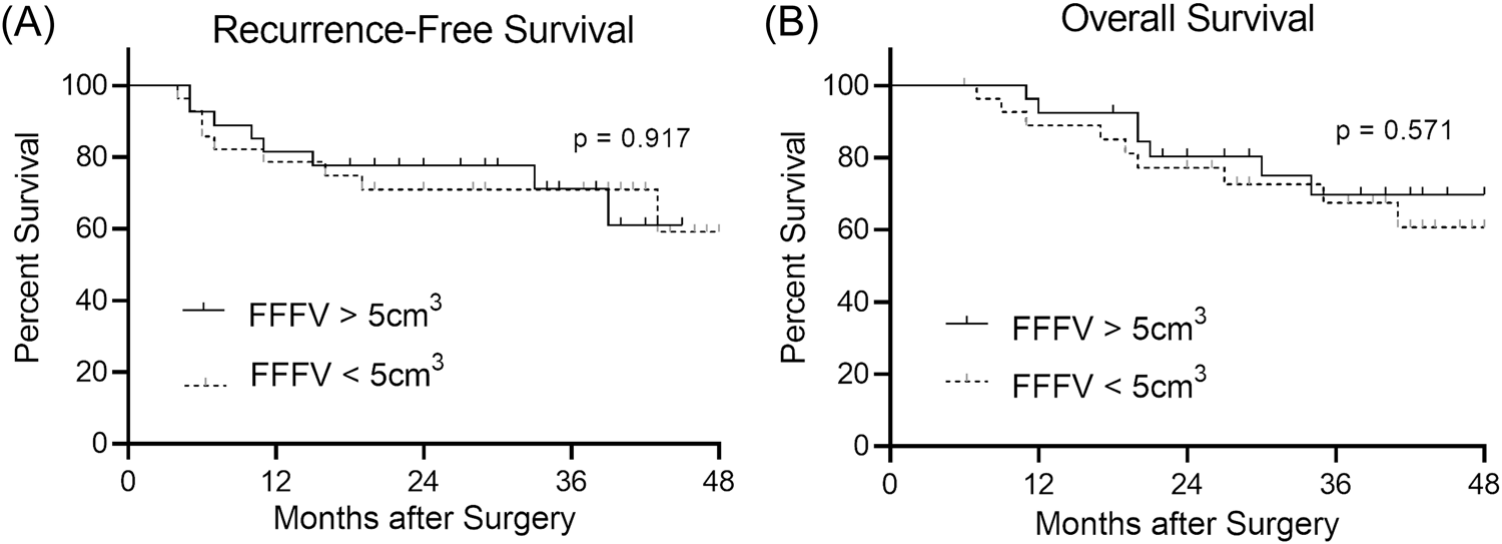
Survival was not different for free flap fat volume (FFFV) above versus below 5 cm^3^. (A) Recurrence‐free survival. (B) Overall survival.

We successfully estimated free flap adipose tissue volume in OSCC patients using LIFEx texture analysis (Figure 1A and B). There was no difference in mean FFFV in cases that recurred versus cases that did not: 13.47 cm^3^ in cancer‐free survivors and 17.99 cm^3^ in cases that recurred (*p* = .56; Figure 2). Kaplan‐Meier survival analyses showed that 2‐year recurrent free survival in patients with high and low FFFV was 61.0% and 59.1%, respectively (*p* = .917; Figure 3A). Two‐year OS was also not significantly different between groups, at 60.6% in the low FFFV group and 69.7% in the high FFFV group (*p* = .571; Figure 3B).

### Wound Healing Complications

**Figure 4:**
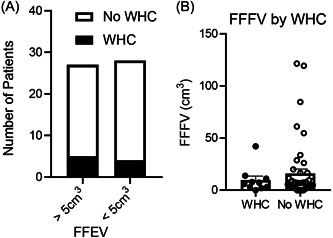
No associations were found between free flap fat volume (FFFV) and wound healing complications (WHC). (A) Incidence of WHC according to FFFV above versus below 5 cm^3^. (B) Measured FFFV between patients with and without WHC. Data are mean + standard error of mean.

Mean FFFV did not differ in patients with wound healing complications versus no complications. Patients with wound healing complications (fistula, abscess, and dehiscence) had a mean FFFV of 9.70 cm^3^, while those who did not have documented complications had a mean FFFV of 16.0 cm^3^ (*p* = .50; Figure 4A). Although our study may have been underpowered to detect effects on wound healing since only 9 patients experienced postoperative wound healing complications, the incidence of wound healing complications between low and high FFFV groups did not demonstrate any identifiable trends. High FFFV patients had a wound healing complication incidence of 18.5%, and that of low FFFV patients was 14.3% (Figure 4B).

## Discussion

We showed that FFFV is not associated with cancer recurrence in OSCC patients undergoing free flap reconstruction. Specifically, we demonstrated that FFFV among patients with recurrent cancer was not statistically different from FFFV among cancer‐free survivors. Moreover, our Kaplan‐Meier analyses show no difference in 2‐year RFS and OS between patients with high and low FFFV.

Our results support previous studies investigating the risk of recurrence in breast cancer patients undergoing autologous fat grafts following surgery. In a recent matched cohort study, Tukiama et al demonstrated that 42 breast cancer patients undergoing autologous fat grafting had no increased risk of locoregional recurrence.^12^ Similarly, Myckatyn and colleagues report in a multicenter study that fat transfer has no association with breast cancer recurrence.^13^ Conversely, Petit et al have shown that only in patients with ductal or lobular intraepithelial breast carcinoma is fat grafting associated with an increased risk of locoregional recurrence.^15^, ^16^ It seems, therefore, that certain tumor subtypes may be more prone to locoregional recurrence following exposure to adipose grafts at the time of surgery than others. In our study, we analyzed patients with OSCC and report no association of FFFV with disease recurrence. However, additional studies investigating multiple head and neck cancer subtypes may produce different results.

Two previous preclinical studies demonstrated increased migration, proliferation, and metastasis of head and neck cancer cells in the presence of ASCs. However, those studies included cell lines used in vitro and murine xenografts, without including any immune cells or an immunocompetent animal model. As such, these studies did not explore the putative immunosuppressive effects of ASCs.^10^, ^11^ Even when immune cells are included, preclinical models do not perfectly represent clinical situations. We hypothesized that ASCs from adipose‐rich free flaps might induce immunosuppression in our surgically treated patients. Rather than study the potential effects of ASCs in preclinical models to then find no effects potentially in the clinical setting, we opted to start by investigating whether the degree of transplanted adipose tissue has any noticeable effects in our patients. Having found no obvious effect in our patients undergoing free flap reconstruction, mechanistic animal studies may not be worthwhile.

Although the primary aim of our study was to investigate the risk of FFFV in OSCC recurrence, given that ASC and adipose tissue have been implicated in wound healing,^3^, ^4^, ^5^ we also compared wound healing complications in high and low FFFV patient groups. Indeed, we observed no trends between FFFV and postoperative fistula formation, abscess, or wound dehiscence. However, only 9 patients experienced such complications. Therefore, a larger study may be needed to confirm our results. Taken together, our results on the effects of FFFV on disease recurrence and wound complications suggest that the head and neck reconstructive surgeon can continue to focus on optimizing form and function when choosing and designing flaps, without concern for the amount of fat content.

Our results must be interpreted considering various study limitations, including the retrospective nature of chart review. Although it is difficult to account for all variables in retrospective studies, we included variables in our chart review that are known to be prognostic predictors in OSCC, including tumor grade, stage, and other comorbid factors. Given that our survival analyses demonstrated no association between FFFV and cancer recurrence or wound healing complications, multivariate analyses were not conducted.

The relatively small sample size may also be considered a limiting factor of our analysis. However, given that our sample size estimate was 120 patients and that no associations were observed in our interim analysis of the 55 patients undergoing surgery in 2017 and early 2018, we believe that the number of patients in our analysis is sufficient to show that there is not a clinically significant difference in recurrence and survival based on FFFV.

Another limitation of this study is the static nature of our FFFV quantification. Previous studies investigating associations of fat content with cancer recurrence have reported fat grafting as a discontinuous variable rather than a quantifiable continuous variable.^12^, ^13^, ^14^ We, however, quantified FFFV on CT imaging acquired in the 6‐ to 12‐month postoperative period. Conceivably, FFFV may fluctuate through time on account of metabolic activity, tissue nutritional demands, and anatomy may be altered following radiotherapy in some cases. Our quantification methodology is only representative of a single point in time.

## Conclusion

In summary, we demonstrated that FFFV is not associated with OSCC recurrence in patients undergoing free flap reconstruction. These findings have significant clinical implications, as preclinical studies continue to investigate the role of ASCs and adipose tissue on the progression of several different cancers. We also reported no association of FFFV with the incidence of wound healing complications. Larger studies investigating multiple head and neck cancer subtypes may be necessary to confirm our findings.

## Author Contributions

**Andre J. Burnham**, conceptualization of the study, collection and analysis of data, drafting and editing of manuscript; **Jaime Wicks**, collection and analysis of data, editing of manuscript; **Kristen L. Baugnon**, conceptualization of the study, editing of manuscript; **Mark W. El‐Deiry**, conceptualization of the study, editing of manuscript; **Nicole C. Schmitt**, conceptualization of the study, collection and analysis of data, drafting and editing of manuscript, supervision of the study.

## Disclosures

### Competing interests

Nicole Schmitt discloses consulting fees from Checkpoint Surgical and Sensorion in addition to research funding from Astex Pharmaceuticals. The remaining authors declare no conflicts of interest.

### Funding source

This research received no external funding.
